# Evaluation of Selected Pro- and Anti-Inflammatory Adipokines in Colostrum from Mothers with Gestational Diabetes Mellitus

**DOI:** 10.3390/ijms26010040

**Published:** 2024-12-24

**Authors:** Jolanta Lis-Kuberka, Marta Berghausen-Mazur, Magdalena Orczyk-Pawiłowicz

**Affiliations:** 1 Division of Chemistry and Immunochemistry, Department of Biochemistry and Immunochemistry, Wroclaw Medical University, M. Skłodowskiej-Curie 48/50, 50-369 Wroclaw, Poland; 2 Department of Neonatology, J. Gromkowski Provincial Specialist Hospital, Koszarowa 5, 51-149 Wroclaw, Poland; 3 Faculty of Medicine, Wroclaw University of Science and Technology, Hoene-Wrońskiego 13c, 58-376 Wroclaw, Poland

**Keywords:** adipokines, gestational diabetes mellitus, human milk, lifestyle diseases

## Abstract

Adipokines related to gestational diabetes mellitus (GDM) are an emerging area of interest. The aim of this study was to evaluate the associations between GDM and adipokine levels in human milk. This was an observational cohort study targeting mothers with gestational diabetes, which evaluated the association of maternal hyperglycemia severity, classified as GDM-G1 (diet treatment) and GDM-G2 (insulin treatment), with colostral adipokines involved in pro- and anti-inflammatory processes. Colostrum was collected from hyperglycemic (N = 34) and normoglycemic (N = 26) mothers, and adipokine levels were determined by immunoenzymatic assay. Among anti-inflammatory adipokines, only for irisin and vaspin, but not for obestatin and adropin, were significantly different levels noted between the GDM-G1, GDM-G2 and non-GDM cohorts. Colostrum of the GDM-G2 subgroup contained more vaspin (4.77 ng/mL) than that of normoglycemic mothers (3.12 ng/mL) and more irisin (26.95 μg/mL) than in the GDM-G1 subgroup (17.59 μg/mL). The levels of pro-inflammatory adipokines, namely, dermcidin, chemerin and visfatin, were at similar levels irrespective of maternal glycemia. Moreover, irisin showed a negative correlation with dermcidin in GDM-G2 and non-GDM cohorts. Associations were observed between colostral irisin and maternal preconception BMI, dermcidin and gestational age, and vaspin and maternal age. This study provides evidence that the way of restoring glucose homeostasis in pregnant women has an impact on the anti-inflammatory adipokines irisin and vaspin, but not on obestatin and adropin. GDM, regardless of severity, did not influence the colostral pro-inflammatory adipokines visfatin, chemerin and dermcidin.

## 1. Introduction

Gestational diabetes mellitus (GDM) is a common pregnancy complication that is associated with impaired glucose metabolism and disturbance of insulin sensitivity, and it can cause serious health problems and, therefore, has significant implications for both maternal and fetal health [1,2,3,4]. The prevalence of pregnancies complicated with GDM increases every year, reaching 15% of the global population (approximately 18 million births annually) [5,6,7,8]. The prevalence of GDM varies across populations and is influenced by factors such as maternal age, ethnicity, obesity and the region of the world [9,10,11,12]. In Europe, it is estimated at 7.8%; however, in developing countries, the occurrence of GDM is significantly higher, with rates of 20.8% in Southeast Asia and up to 27.6% in the Middle East and North Africa [7]. 

In Poland, according to IADPSG and WHO criteria, the diagnosis of GDM is based on values of the oral glucose tolerance test (OGTT) at 24–28 weeks or earlier. In 2011, the World Health Organization as well as the American Diabetic Association accepted glycated hemoglobin (HbA1c) as a diagnostic tool for diagnosing GDM [13,14]. HbA1c reflects average plasma glucose over the previous three months and it is used for monitoring glycemic control in patients with diabetes (HbA1c ≥ 6.5% or 48 mmol/mol) diagnosed before pregnancy (HbA1c correlates very well with the risk of diabetes complications) [15]. It was well established that the poor glycemic control of pregnant women has adverse maternal and neonatal outcomes [1,2,3,15,16,17,18,19,20], but appropriate antenatal care can help mitigate risks associated with diabetes and ensure maternal well-being and the proper development of the fetus and newborn [21,22,23,24,25,26,27,28,29,30,31,32,33,34,35,36]. 

The molecular events responsible for the development of GDM are not fully understood [37,38]. However, early pregnancy excessive weight gain might be related to adipocyte hypertrophy and such a state may contribute to fat accumulation and favor shifting the balance between anti-inflammatory (M2 phenotype) and pro-inflammatory (M1 phenotype) macrophages toward the M1 state [39,40,41,42,43]. The consequence of such a state is the increased involvement of adipocytes in the synthesis of pro-inflammatory cytokines and adipokines, inducing systemic, low-grade inflammation, which accompanies insulin resistance, diabetes and metabolic disorders associated with obesity [44,45,46,47]. 

In general, adipokines can be classified as those promoting inflammation (i.e., leptin, resistin, chemerin and visfatin) and those with anti-inflammatory properties (i.e., adiponectin, ghrelin, irisin, obestatin, adropin, vaspin and dermcidin) [48,49,50,51]. While pro-inflammatory adipokines stimulate inflammation, the anti-inflammatory adipokines help mitigate the chronic low-grade inflammation associated with obesity, diabetes and metabolic disorders [47,50,51]. The anti-inflammatory actions of adipokines include suppression of inflammation by blocking the NF-κB signaling pathway [51,52,53] and thereby reducing the levels of pro-inflammatory cytokines such as TNF-α and IL-6 [49,51]. Among the well-characterized adipokines is the group of hormones classified as appetite-regulating adipokines (adiponectin, leptin, resistin and ghrelin) [54,55,56,57,58]. Knowledge about other adipokines is systematically expanding every year, and it was reported that obestatin, irisin and adropin are molecules involved in energy metabolism and homeostasis [59,60,61]. Vaspin and visfatin are closely linked to metabolic disorders [62,63,64], while dermcidin and chemerin have antimicrobial properties, but they are also related to insulin resistance and inflammation [65]. 

Diabetes is accompanied by increased levels of leptin [66], resistin [67], chemerin [68], visfatin [69] and vaspin [64,70,71] and decreased levels of adiponectin [72], ghrelin [73], irisin [74], obestatin [75], adropin [76,77] and dermcidin [78]. Moreover, it was found that chemerin, visfatin, vaspin, irisin and dermcidin are correlated positively, while obestatin and adropin negatively, with BMI (body mass index) and HOMA-IR (homeostatic model assessment for insulin resistance) [60,79,80,81]. Some studies reported that higher levels of circulating adiponectin are associated with a lower risk of type 2 diabetes [82], GDM and adverse perinatal outcomes [83,84]. The mechanism of action of adiponectin is related to improving insulin sensitivity and modulating glucose and fatty acid catabolism. An opposite effect to that of adiponectin is exhibited by chemerin [84,85,86], which inhibits insulin signaling and glucose catabolism. The authors [84,87,88,89,90] hypothesized that a high ratio of chemerin to adiponectin might be crucial for dyslipidemia and metabolic syndrome in persons with impaired metabolism. Vaspin and visfatin are closely associated with insulin resistance [62,63], but the data indicate that they act in two different directions [91]. Vaspin shows insulin-sensitizing effects, and a high level of it is associated with a better prognosis. On the other hand, visfatin concentration is strongly correlated with HbA1c, and in patients with type 2 diabetes, it reflects poor glycemic control and is associated with poor prognosis [91]. 

The shifts in adipokine levels in the maternal circulation can translate into disturbances in the development of the fetus, and after delivery, the changes in their concentrations in milk might have an impact on the postnatal growth of offspring [92,93,94,95,96,97,98]. Milk adipokines may contribute to the reduced risk of development of obesity and diabetes in childhood and adulthood [99,100]. The reduced level of irisin in human milk may have negative effects on infant weight and lipid regulation [101], while Mól and coworkers [102] reported that altered concentrations of irisin and visfatin in milk from preterm delivered mothers might eventually lead to a higher risk for metabolic disorders in prematurely born children in later years. Other milk hormones, such as chemerin and dermcidin, were discovered relatively recently, and little is known about their changes in relation to maternal metabolic disorders. However, they are involved in the protection of infants from infections [65]. So far, the presence of vaspin in human milk has not been the subject of research, but this insulin-sensitizing adipokine has been detected in maternal and cord plasma [103,104].

There is a large knowledge gap regarding the evaluation of the concentration and importance of adipokines in human milk in relation to maternal health status. In light of the above, the aim of this study was the evaluation of associations between GDM and adipokine levels in human milk. So far, none of the above-mentioned adipokines, namely, chemerin, irisin, visfatin, vaspin, dermcidin, obestatin and adropin, have been evaluated in relation to managing maternal hyperglycemia during pregnancy classified as GDM-G1 (diet-treatment GDM) and GDM-G2 (insulin-treatment GDM). Moreover, we evaluated associations of maternal, obstetrical and neonatal factors on the profile of colostral anti- and pro-inflammatory adipokines.

## 2. Results

### 2.1. Characteristics of the Study Population

Sixty milk samples were collected from lactating normoglycemic women (N = 26) and women exposed to gestational diabetes mellitus (N = 34). The characteristics of participants are outlined in Table 1. The colostrum samples were collected from white Europeans and the analyzed cohorts did not differ in maternal anthropometric (pre-pregnancy BMI, age), obstetric (gestational age, mode of delivery and lactation day) or neonatal (birth weight, gender, Apgar score) parameters that could affect hormone concentration in colostrum. 

The mean maternal age was 33.00 ± 4.81 years and pre-pregnancy BMI was 23.99 ± 4.81 kg/m^2^; no significant differences were found among the analyzed cohorts of mothers in the GDM-G1, GDM-G2, and non-GDM groups. The great majority of the analyzed subgroups of women delivered their newborns at term, namely, at 38–41 weeks of gestation (GDM-G1: 76.47%, GDM-G2: 70.59%, and non-GDM: 84.62%) (Table 1). Almost three-quarters of recruited mothers delivered by cesarean section (73.33%), and the distribution of this variable was as follows: GDM-G1: 76.47%, GDM-G2: 70.59%, and non-GDM: 84.62%, respectively.

The neonatal birth weight did not differ among groups and was 3238.82 ± 587.37 g, 3187.94 ± 484.11 g, and 3337.31 ± 571.26 g, respectively, for GDM-G1, GDM-G2 and non-GDM (Table 1). Male newborns made up 58.82%, 52.94% and 26.92% of the GDM-G1, GDM-G2 and non-GDM groups, respectively; however, no information was recorded for 6.67% (Table 1). Newborn’s condition expressed as the Apgar score was at similar level, at least nine points.

### 2.2. Concentration of Colostral Adipokines in Relation to Maternal Hyperglycemia (GDM vs. Non-GDM)

The analyzed colostral adipokine concentrations, namely, for irisin, adropin, obestatin, vaspin, visfatin, dermcidin and chemerin, did not show a statistically significant difference between hyperglycemic (GDM) and normoglycemic (non-GDM) mothers (Table 2). The median value for chemerin and obestatin concentrations was slightly lower, but not significantly, in the GDM group (0.90 ng/mL and 0.28 ng/mL, respectively) in comparison to the non-GDM group (1.26 ng/mL and 0.30 ng/mL, respectively) (Table 2). By contrast, in the group of mothers with GDM, irisin, adropin and dermcidin (19.87 µg/mL, 0.49 ng/mL, and 136.55 ng/mL, respectively) showed slightly higher, though not significantly, median values in comparison to the normoglycemic cohort (18.53 µg/mL, 0.34 ng/mL, and 124.70 ng/mL, respectively) (Table 2). Similarly, the median values for visfatin and vaspin were higher, but not significantly, for the GDM group (1.38 ng/mL and 4.69 ng/mL, respectively) than for the non-GDM group (1.30 ng/mL and 3.12 ng/mL, respectively) (Table 2). 

### 2.3. Concentration of Colostral Adipokines in Relation to Severity of Maternal Hyperglycemia

**Figure 1:**
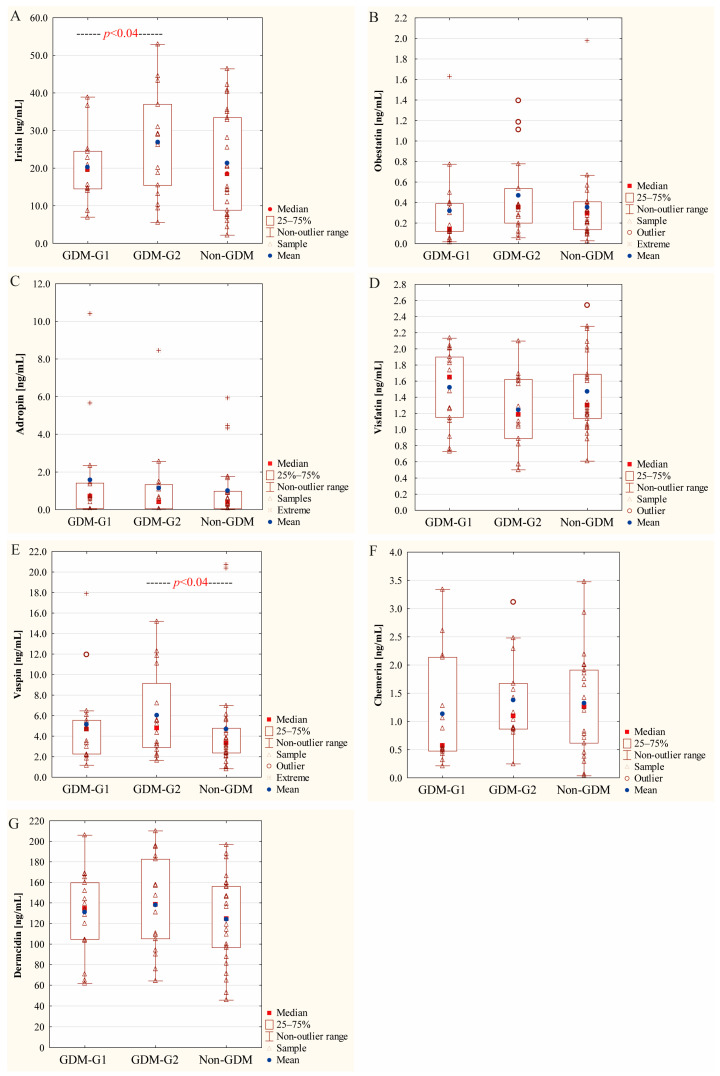
Comparison of irisin (**A**), adropin (**B**), obestatin (**C**), visfatin (**D**), vaspin (**E**), chemerin (**F**) and dermcidin (**G**) concentrations in colostrum between gestational diabetic (G1 and G2) and normoglycemic (non-GDM) mothers. Data are given as mean and median values and 25th and 75th quartiles. A *p*-value lower than 0.05 was regarded as significant.

Among analyzed adipokines, the concentrations of irisin and vaspin differed significantly in relation to the severity of maternal glycemia classified as GDM-G1 (diet treatment) and GDM-G2 (insulin treatment). The median value of irisin for GDM-G1 was significantly lower (17.59 μg/mL) in comparison to GDM-G2 (26.95 μg/mL) (*p* = 0.04), but not to the non-GDM (18.53 μg/mL) group. On the other hand, the concentration of colostral vaspin was significantly higher in the GDM-G2 subgroup (4.77 ng/mL) than in the non-GDM group (3.12 ng/mL) (*p* = 0.04); however, no significant difference between the GDM-G1 (4.11 ng/mL) and GDM-G2 cohorts (*p* = 0.31) was found (Figure 1, Table 2). 

Although no significant differences were noted for obestatin, the median value for the GDM-G1 subgroup was lower (0.14 ng/mL), but not significantly, than values observed for GDM-G2 (0.36 ng/mL) and non-GDM (0.30 ng/mL) cohorts, respectively (Figure 1, Table 2). The cut-off for statistical significance was set at a *p*-value lower than 0.05, but for obestatin, a borderline *p*-value equal to 0.05 was obtained. 

In contrast to irisin and vaspin, the concentrations of adropin and dermcidin were not significantly affected by maternal glycemic state (*p* > 0.05). The median values of adropin and dermcidin were 0.70 ng/mL and 134.51 ng/mL for GDM-G1, 0.42 ng/mL and 138.58 ng/mL for GDM-G2, and 0.34 ng/mL and 124.70 ng/mL for the non-GDM group, respectively. 

Similarly, the median value of chemerin and visfatin concentrations did not differ significantly in relation to the severity of hyperglycemia, namely, for GDM-G1, 0.57 ng/mL and 1.65 ng/mL; for GDM-G2, 1.10 ng/mL and 1.19 ng/mL; and for non-GDM, 1.26 ng/mL and 1.30 ng/mL, respectively (Figure 1, Table 2).

### 2.4. Correlations Between Adipokines and Maternal, Obstetrical and Neonatal Outcomes 

**Figure 2:**
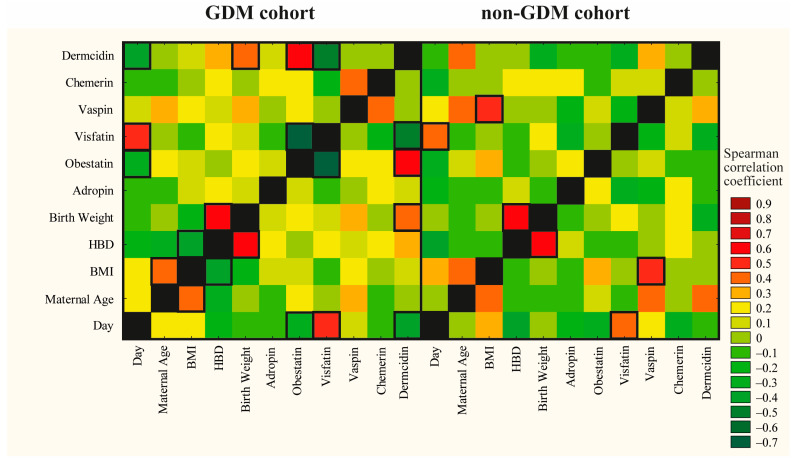
Correlations between the concentration of adipokine in milk collected from GDM and non-GDM mothers and day of lactation, age, preconceptional BMI, and week of gestation. The Spearman correlation coefficient is represented in the heat map following the color in the legend. BMI—preconceptional body mass index; HBD—week of gestation. Bold frames represent correlations with statistical significance (*p* < 0.05).

To assess the influence of day of lactation, maternal age, preconception BMI, gestational age and birth weight of newborns on analyzed adipokine concentrations in colostrum samples, Spearman correlation coefficient values were estimated (Figure 2, Supplementary Table S1). Due to the lack of significant differences for colostral adropin, obestatin, visfatin, vaspin, chemerin and dermcidin levels between GDM-G1 and GDM-G2 mothers, these two cohorts were merged together.

Regardless of maternal glycemic state, weak positive correlations (GDM: r = 0.43 and non-GDM: r = 0.40, *p* < 0.05) between visfatin levels and day of lactation were observed. Additionally, in the GDM cohort, but not in the non-GDM group, weak negative correlations of dermcidin and obestatin levels with day of lactation (r = −0.42 and r = −0.36, *p* < 0.05, respectively) were noted (Figure 2, Supplementary Table S1). On the other hand, for adropin, vaspin and chemerin levels, no correlation with day of lactation was found.

In contrast to the GDM cohort, for the non-GDM group, a weak positive correlation (r = 0.43, *p* < 0.05) was found between vaspin concentration and maternal BMI. None of the analyzed adipokines showed a significant correlation with maternal age (Figure 2, Supplementary Table S1). In both groups, namely, GDM and non-GDM, no significant correlations between levels of analyzed adipokines and gestational age were found (Figure 2, Supplementary Table S1). The dermcidin concentration, but not obestatin, adropin, visfatin, vaspin or chemerin, showed a weak correlation (r = 0.37, *p* < 0.05) with birth weight, but in the GDM group only. 

The analysis of Spearman correlation coefficient values for colostral adipokine concentrations showed that in the GDM group, there was a moderate negative (r = −0.54, *p* < 0.05) relationship between dermcidin and visfatin levels. Moreover, the concentration of colostral obestatin, but from the GDM group only, showed a moderate positive correlation with dermcidin (r = 0.56, *p* < 0.5) and a moderate negative correlation with visfatin (r = −0.71, *p* < 0.5) (Figure 2, Supplementary Table S1). 

### 2.5. Human Colostral Irisin Levels Reflect Maternal Health State

Due to the statistically significant difference observed for irisin level between GDM-G1 and GDM-G2 mothers, the cohorts were analyzed separately.

To evaluate the relationship between maternal age, preconception BMI, the week of gestation, neonatal birth weight and irisin concentration in colostrum, with an emphasis on maternal hyperglycemic state, Spearman’s correlation values were calculated (Table 3).

In the GDM-G2 and non-GDM cohorts, but not in the GDM-G1 subgroup, moderate negative correlations (r =−0.51 and r = −0.53, respectively, *p* < 0.05) were found between dermcidin and irisin levels. Additionally, only in the non-GDM group, weak positive correlations between irisin level and maternal BMI (r = 0.45, *p* < 0.05) and between irisin level and newborn birth weight (r = 0.40, *p* < 0.05) were observed.

### 2.6. Associations of Maternal, Obstetrical and Neonatal Factors with Colostral Adipokines

To evaluate the associations of maternal, obstetrical and neonatal factors with colostral adipokines, multiple regression analysis was performed.

Taking into account maternal outcomes, associations between irisin levels and maternal preconception BMI (β = 0.40; 95% CI 0.04–0.76; *p* < 0.04) were noted. Additionally, the vaspin levels in colostral samples showed an association with maternal age (β = 0.55; 95% CI 0.25–0.86; *p* < 0.0008) (Table 4). No associations were found between milk hormones, namely, irisin, obestatin, adropin, vaspin, visfatin, chemerin and dermcidin, and maternal hyperglycemia during pregnancy (Table 4).

On the other hand, taking into account obstetrical outcomes, an association between dermcidin level and gestational age (β = 0.45; 95% CI 0.02–0.88; *p* < 0.04) was found. No associations were observed for analyzed colostral adipokines and delivery mode (Table 5). 

No associations of the neonatal factors such as birth weight and Apgar score with the concentrations of colostral adipokines, namely, chemerin, irisin, visfatin, vaspin, obestatin, dermcidin and adropin, were detected (Table 5).

## 3. Discussion

The health of women before and during pregnancy has a critical role in shaping the optimal fetal development and well-being of newborns and infants. Gestational diabetes mellitus is a complex metabolic disease, which affects glucose homeostasis during pregnancy, and it translates into adverse maternal, fetal and neonatal outcomes. The uncontrolled glucose level is associated with adipocyte and macrophage activation, which supports an imbalance between anti- and pro-inflammatory adipokine levels in maternal plasma. In contrast, studies examining adipokine levels in human colostrum in relation to the severity of maternal hyperglycemia during pregnancy are limited. Milk adipokines are crucial molecules that link maternal metabolic status and early infant development. This study provides evidence that the way of restoring glucose homeostasis in pregnant women has an impact on anti-inflammatory adipokines, such as irisin and vaspin, but not on obestatin and adropin. In contrast, the treatment of GDM with different severity levels did not leave a mark on the colostral pro-inflammatory adipokines, namely, visfatin, chemerin and dermcidin.

Among the four analyzed anti-inflammatory adipokines (irisin, vaspin, obestatin and adropin), only for irisin and vaspin were some differences related to managing glucose imbalance during pregnancy found. Glucose imbalance during pregnancy translates into plasma irisin level, but these changes are not reflected in cord plasma [105,106,107] or, as was shown in this study, in colostrum. Although a significant difference was detected for irisin level between the GDM-G1 (17.59 μg/mL) and GDM-G2 (26.95 μg/mL) subgroups, our study did not reveal a significant difference between GDM and non-GDM mothers, unlike the results reported previously by Aydin et al. [108] and Fatima et al. [109]. Despite that it was reported that plasma irisin levels increase transiently during and shortly after bouts of exercise [110,111], in our study, significantly higher irisin levels for colostrum of GDM-insulin treated mothers 26.95 μg/mL in comparison to colostrum of mothers, who implemented lifestyle intervention, including diet and moderate physical activity (GDM-G1 cohort) 17.59 μg/mL, were demonstrated. The observed differences in irisin levels between GDM-G1 and GDM-G2 cohorts might result from implementing the more effective restoration of glucose homeostasis; however, it should be noted that irisin levels might also be affected by anthropometric parameters, body composition and metabolic profiles of the mother [112]. Milk irisin may have an impact on the adaptation of newborns for postnatal life with respect to thermoregulation, regulation of infant weight, and energy metabolism and homeostasis [101,102]. In this study, but only for normoglycemic mothers, we observed moderate correlations of colostral irisin with maternal preconception BMI (r = 0.45; *p* < 0.05), and also with birth weight (r = 0.40; *p* < 0.05). Our observations are in line with data for maternal serum [109] and cord blood plasma [113]. Additionally, the lack of correlation with maternal and gestational age shown in our study is consistent with previous reports [105,114,115]. However, multiple regression analysis showed that colostrum irisin levels increased with BMI independently of maternal glycemic status, which is in line with circulating irisin in obese individuals compared to healthy controls [116].

Similar to irisin, for vaspin, significant differences between GDM and non-GDM cohorts were not revealed; however, a higher concentration in colostrum samples of insulin-treated mothers (4.11 ng/mL) than normoglycemic women (3.12 ng/mL) was observed. In an animal model, vaspin level is significantly higher in obese mice with insulin resistance [117] similar to our study group of mothers with GDM insulin-treated. Based on the above, it might be suggested that the observed differences between analyzed sub-cohorts are linked with the insulin-sensitizing properties of vaspin, and thus its elevated level in human milk might be explained as a compensatory mechanism against maternal insulin resistance during hyperglycemia-affected pregnancy. To the best of our knowledge, this study is the first to characterize colostral vaspin in relation to maternal hyperglycemia. Hernández-Rodríguez and coworkers [104] reported that the increase in fetal vaspin concentration in response to maternal hyperglycemia was related to improved fetal insulin utilization. Also, a recent study [79] suggested that an elevated serum vaspin level may imply its compensatory role against metabolic disorders in obese patients. In this study, we did not reveal a significant difference (*p* = 0.06) for the GDM overall cohort in comparison to the non-GDM group; the *p* value was on the borderline of significance, and it was not possible to reach a definite conclusion—similar to the inconsistent data for serum vaspin [62,103,118,119,120]. In this study, a moderate positive correlation (r = 0.43, *p* < 0.05) was found for vaspin and maternal BMI, but only for normoglycemic mothers. Independently of maternal glycemic status, the multiple regression analysis revealed a positive association between colostrum vaspin level and age of women, and this is in line with the data presented by Xu and coworkers [121]. 

It is considered that obestatin acts as a compensatory mechanism in response to inflammatory conditions [122]. The management of glucose imbalance during pregnancy did not significantly affect colostral obestatin levels, namely, GDM-G1: 0.14 ng/mL, GDM-G2: 0.36 ng/mL (GDM: 0.28 ng/mL) and non-GDM: 0.30 ng/mL. The lack of statistically significant differences for obestatin levels in the analyzed cohorts is in line with data presented previously [123] for the serum of healthy and diabetic pregnant women. However, it should be emphasized that the *p* value for the GDM-G1 and GDM-G2 subgroups was of borderline significance (*p* = 0.05). In contrast, in obese and diabetic individuals, a significant decrease in the concentration of serum obestatin was observed [12,124]. Moreover, our study did not reveal the existence of significant correlations between colostral obestatin and maternal anthropometric parameters (BMI and age) or obstetrical variables, such as gestational age and neonatal birth weight. The possible function/role attributed to milk obestatin is the participation in appetite-regulating processes, including suppressing appetite and reducing food intake [125,126]. In animal models, exogenous sources of obestatin stimulate the exocrine pancreas secretion via an indirect vagal mechanism [127], and thus this adipokine might prevent diet-related metabolic disorders. So far, the impact of human milk obestatin on offspring’s postnatal development has not been demonstrated, and further analysis in this area is needed. Słupecka-Ziemilska and coworkers [128] reported that one of the possible directions is the evaluation of the ghrelin/obestatin ratio, which is considered a crucial factor for modulating energy homeostasis and adaptation of the organism to nutritional challenges. Moreover, the protective and healing effects of obestatin, due to the improvement in blood flow, improved cell vitality and proliferation, and a reduced expression of IL-1β and TNF-α was suggested [122,129,130]. These properties might be especially valuable for the perinatal care of offspring born prematurely with the immaturity of the intestinal epithelial barrier. In an animal model of necrotizing enterocolitis, obestatin found in rat’s milk reduced intestinal damage and prevented necrosis through anti-inflammatory and antiapoptotic effects [131].

Another important player in diabetes and obesity, classified as an anti-inflammatory adipokine, is adropin, higher values of which may be a compensatory mechanism that serves to prevent insulin resistance and improve glucose tolerance [132,133]. In this study, the level of colostral adropin was not affected by the severity of maternal hyperglycemia (GDM: 0.49 ng/mL; non-GDM cohorts 0.34 ng/mL; GDM-G1: 0.70 ng/mL and GDM-G2: 0.42 ng/mL). However, Aydine and coworkers [108] noted a significantly lower level of adropin in GDM (8 ng/mL) than in non-GDM colostrum samples (18 ng/mL), but the size of the analyzed groups was smaller than in our study. Additionally, the adropin level in the circulation of women with metabolic disorders did not differ at 27–32 weeks of gestation, but a significant decrease was noted in morbidly obese and obese women at 37–39 weeks of gestation [134]. On the other hand, the maternal serum/plasma adropin concentrations were significantly higher in women with exposure to GDM as compared to non-GDM groups [135], and according to Beigi et al. [136], adropin might serve as an independent predictor of GDM. It is interesting that in an animal model with overexpressed adropin, a high-fat diet did not demonstrate the clinical manifestation of obesity and glucose imbalance [137,138], which was explained by the beneficial effect of adropin on sensitization of insulin signaling pathways, and improved glucose metabolism. Due to limited research and contradictory data, a definite conclusion cannot be established. However, it should be pointed out that adropin levels might be affected by diets and some functional foods [139] such as flavonoids—myricetin [140,141]—fructose [142] and fish oil (unsaturated fatty acids), which can significantly increase blood adropin levels [139,141]. Similarly, probiotics have shown the potential to promote the secretion of adropin [143]. Therefore, further investigations of this area with detailed information about maternal diet during pregnancy are needed. In this study, there was no statistically significant correlation between the concentration of colostral adropin and maternal BMI, which is in line with data presented previously [134] for adropin concentrations in serum.

In contrast to the anti-inflammatory adipokines irisin and vaspin, the pro-inflammatory adipokines visfatin (GDM-G1: 1.65 ng/mL, GDM-G2: 1.19 ng/mL and non-GDM: 1.30 ng/mL), dermcidin (GDM-G1: 134.51 ng/mL, GDM-G2: 138.58 ng/mL and non-GDM: 124.70 ng/mL) and chemerin (GDM-G1: 0.57 ng/mL, GDM-G2: 1.10 ng/mL and non-GDM: 1.26 ng/mL) were not affected by GDM regardless of its severity. Although the observed values for visfatin are lower than those reported by other authors [102,144], its presence in human colostrum might prevent weight loss during the early stage of postnatal life, and it might result in a visfatin-mediated insulin-like effect on offspring’s adipose tissue. Our study revealed no correlations between colostral visfatin and maternal BMI or newborn’s birth weight. Currently, research is underway on the role of visfatin and omentin-1 as markers of the nutritional status of newborns born to diabetic mothers [145]. 

In contrast to our data for colostral chemerin, Ustebay and coworkers [65] observed significantly higher values for the GDM than for the non-GDM cohort and suggested protection of infants from infections during infancy. However, this adipokine is also associated with insulin resistance, which might be linked with altered levels of cleaved and degraded chemerin [89]. The analysis of correlations between chemerin, fasting insulin and HOMA-IR values provided conflicting results [146]. Moreover, detection of chemerin levels in plasma samples, placenta and adipose tissues from mothers with GDM also presented contradictory results, which were summarized in a review by Gutaj et al. [146] and Lis-Kuberka et al. [97]. Recently, new insights into the role of chemerin in the maternal–infant dyad were demonstrated by Fatima and coworkers [147], who reported the association of the chemerin gene promoter methylation in maternal blood and breast milk during gestational diabetes and suggested its possible role in contributing to childhood obesity.

So far only one report has evaluated dermcidin levels in human milk [65] and, in contrast to our data, noted that its level significantly increased with GDM. As reported previously [65,148,149], dermcidin may contribute to the protection of newborns and infants from infections. Independently of maternal glycemic status, multiple regression analysis revealed a positive association between colostrum dermcidin levels and gestational age, and this is in line with data presented by Ustebay and coworkers [65]. We hypothesized that the found correlation might be related to the transfer of elements that support the immature immunity system of newborns. In light of the above, it seems possible that dermcidin in human milk supports the immune system of breastfed newborns and contributes to the protection of infants from infections during infancy [65,150]. Nevertheless, the molecular mechanism of dermcidin’s effect on the neonate’s immature system remains unclear.

Adipokines form a network of interconnected molecules important for the integration of systemic metabolism with immune function [51,151] and might serve as indicators in pathological states. In this report, a moderate negative correlation was found between irisin and dermcidin levels, in both GDM-G2 and non-GDM groups (r = −0.51 and r = −0.53, *p* < 0.05). However, a similar relationship was not observed for the diet-treatment group (r = 0.17, *p* > 0.05). For hyperglycemic mothers, significant associations between dermcidin and obestatin (r = 0.56, *p* < 0.05) and between dermcidin and visfatin (r = −0.54, *p* < 0.05) were found. Moreover, this study indicates the existence of a moderate correlation between colostral visfatin and obestatin (r = −0.71, *p* < 0.05). It is worth emphasizing that high levels of visfatin and low levels of obestatin were previously reported by Putera and coworkers [152] for obese but not for normoglycemic mothers, and it was associated with increased beta pancreas cell dysfunction and insulin resistance. 

### Strength and Limitations

The undeniable strength of the present study is the evaluation of colostral adipokine levels in milk samples in relation to the severity of maternal hyperglycemia, which is the first such detailed analysis. Further strengths of the study include the standardized sample collection protocol and a very homogeneous cohort of lactating women with respect to maternal and obstetric outcomes, which limited potential confounding factors. Moreover, the evaluation of the adipokine profile in relation to covariates such as BMI and age of the mother, week of gestation, neonatal birth weight, Apgar score and mode of delivery is an added advantage. Presented, data were compared with milk/plasma adipokine levels, which were measured by enzyme-linked immunosorbent assay, and it is therefore advantageous in this study. The second method used for adipokine determination was radioimmunoassay (RIA); however, these data were not compared with our results, due to the different methodological approaches. Additionally, it is worth pointing out that the ELISA kits used for the determination of adipokine levels in human colostrum were validated. At the same time, the two most important things were refined, namely, colostrum sample dilution optimization and adjusting the range of calibration curves to fit the colostral adipokine concentrations, and this was the strength of this study. According to the scientific databases, namely, Web of Science and National Center for Biotechnology Information, the comparison of a performed-by-us validation procedure with other analyses is particularly difficult due to the lack of information on the validation stage of the assay as well as on the sample dilutions used.

However, the following limitations should be noted when interpreting the results. Small sample sizes in our study introduce several limitations, including limited generalizability, the risk of random variability, slightly lower precision and reliability levels, and limited exploration of heterogeneity. However, we applied a protocol for selecting participants of the study, which allowed us to obtain a similar distribution of maternal age, BMI and gestational age in the analyzed cohorts, to minimize bias. Moreover, the published literature on adipokine levels in human milk, especially for colostrum, is limited and incomplete, especially in regard to milk from mothers with GDM. Secondly, our relatively small groups of colostrum samples consisted only of white European women, which may limit the extrapolation of our data to a wider population of women and/or different geographical zones. Jara et al. [153] reported that race might have effects on maternal serum adiponectin and leptin levels during pregnancy and it seems possible that these alterations can translate also into other adipokine levels in human milk. Previously, Ruiz and coworkers [154] analyzed the immune profile of human milk from healthy women living in different geographical and socioeconomic settings and concluded that there is substantial variation within and, particularly among, human subpopulations. In light of the above, it is possible that the observed differences are associated with the European population. Additionally, more detailed information is needed about GDM mothers’ lifestyle intervention, including diet and physical activity. Our study was based on the general classification of pregnant women as a GDM-G1 or GDM-G2, and the medical interview did not contain detailed questions about dietary habits and daily physical activity, while it was reported that even moderate intensity increases anti-inflammatory adipokines and improves diabetes status [155,156,157]. Moreover, lack of information regarding the value of glycated hemoglobin (HbA1c) might be considered a limiting factor, namely, it cannot be ruled out that in the analyzed cohort were asymptomatic women with undiagnosed diabetes. Finally, the study was observational, and a direct causal association between maternal GDM, colostral adipokine profile, and offspring anthropometric parameters cannot be established. Due to the above, the impact of maternal exposition on GDM during pregnancy on colostral adipokine levels cannot be unequivocally stated. Future, larger studies will provide stronger and more reliable results due to smaller margins of error and lower standards of deviation.

A comparison of the present data with other analyses is difficult due to the limited amount of research, as well as discrepancies in human milk sampling, and changes related to lactation stages and health of women during pregnancy, childbirth and the postnatal period [153,154]. Addressing these issues could further explain the role of milk adipokines in the metabolic programming of offspring born by mothers whose pregnancy was complicated by hyperglycemia. In the future, the emphasis should be on expanding the sample size and conducting multi-center studies to better understand the role of human milk adipokines in the growth and well-being of newborns and infants.

## 4. Materials and Methods

### 4.1. Study Designed

This study was a retrospective observational cohort study targeting patients with gestational diabetes who delivered at the First Department and Clinic of Gynecology and Obstetrics, Wroclaw Medical University (Wroclaw, Poland). To calculate the sample size (calculated using StatSoft, Inc., Tulsa, OK, USA), we assumed a 95% confidence level (α error of 0.5%), and a 5% loss rate; a sample of 69 subjects was required. Of the 98 mothers, we excluded women who had multiple pregnancies, chromosomal abnormalities, preterm birth (<35 weeks), uncontrolled thyroid disease, epilepsy or diabetes type I and II, alcohol consumed and cigarette smoke during pregnancy, and incomplete data (age, BMI, gestational age, newborns weight). Initially, the number of samples qualified for the study was higher, namely, 72; however, due to the donation of a relatively small sample volume (after meeting the newborn’s nutritional needs), the colostrum volume was not sufficient to perform the necessary defatting procedure as well as determinations for all adipokines in this study. Finally, sixty breastfeeding mothers participated in the study.

### 4.2. Recruitment of Breastfeeding Mothers

In the research, women with GDM (hyperglycemic group; GDM group) and lactating normoglycemic mothers (non-GDM group), who received postpartum care in the First Department of Gynecology and Obstetrics, Wroclaw Medical University (Wroclaw, Poland), were selected. The enrolling criteria of women with GDM were an abnormal fasting blood glucose level and/or oral glucose tolerance test (OGTT) following ingestion of 75 g of glucose [58,158,159,160,161].

The health status of the mother and infant was noted and included the following data: women’s race, age and preconception BMI, week of gestation, mode of delivery, newborn’s birth weight, newborn’s gender and Apgar score at 5 min. The exclusion criteria included cigarette smoking and alcohol consumption during pregnancy, multiple pregnancies, preterm delivery before 35 weeks of gestation, genetic defects of the fetus, and neonatal weight not appropriate for gestational age. The group of women with hyperglycemia during pregnancy was divided into two subgroups: diet-treatment GDM (GDM-G1 cohort) and insulin-treatment GDM (GDM-G2 cohort) [58,158,159,160,161]. Overall, 60 women were enrolled in the study (GDM-G1: N = 17, GDM-G2: N = 17 and non-GDM: N = 26).

### 4.3. Ethics

The Ethics Committee at Wrocław Medical University approved the study (approval code: KB-200/2023N; approval date: 31 October 2023) and informed written consent was obtained from all mothers enrolled for the research. The study complied with Good Clinical Practice and the Declaration of Helsinki.

### 4.4. Colostrum Collection

The colostrum samples from the 1st to 7th days of lactation were collected from each woman at the First Department and Clinic of Gynecology and Obstetrics, Wroclaw Medical University. Lactating women, after breakfast, provided milk samples in a time interval from 08:00 to 12:00. After the collection procedure, the colostrum samples were frozen at −20 °C [58,161].

### 4.5. Sample Pre-Treatment for Analysis

All collected colostrum samples were centrifuged at 3500× *g* at 4 °C for 35 min to obtain the aqueous phase of milk without fat and cells that interfere with the analysis. The obtained aqueous phase of colostrum samples was stored at −20 °C [58,161].

### 4.6. Determination of Adipokine Concentrations

Colostral levels of irisin, obestatin and adropin were measured using a commercially available ELISA kit (orb550756, orb563462 and orb780287, respectively; Biorbyt, Cambridge, United Kingdom) after previous adaptation of the assay for determination in milk samples. The detection ranges of the ELISA were 1.6–100.0 ng/mL, 0.02–1.0 ng/mL and 0.03–2.0 ng/mL, respectively. All defatted colostrum samples were assayed in duplicate. Colostrum samples were diluted 500-fold, 2-fold and left undiluted, for irisin, obestatin and adropin, respectively. The intra-assay and inter-assay coefficients of variation were for irisin 2.8% and 8.5%, for obestatin 3.1% and 5.3%, and for adropin 4.5% and 9.3%, respectively.

Colostral levels of visfatin and vaspin were measured using a commercially available ELISA kit (orb442894 and orb442869, respectively; Biorbyt, Cambridge, UK) after previous adaptation of the assay for determination in milk samples. The detection ranges of the ELISA assays were 0.02–1.0 ng/mL and 0.2–10.0 ng/mL, respectively. The colostrum samples were diluted 20-fold for the determination of visfatin concentration and 2-fold for the determination of vaspin concentration. The defatted colostrum samples were assayed in duplicate. The intra-assay and inter-assay coefficients of variation were for visfatin 2.2% and 6.3% and for vaspin 2.5% and 6.1%, respectively.

Colostral levels of dermcidin and chemerin were measured by an ELISA commercial kit (orb564791 and orb865301 respectively; Biorbyt, Cambridge, UK) after previous adaptation of the assay for determination in milk samples. The detection range of the ELISA assays was 0.3–20.0 ng/mL and 0.08–5.0 ng/mL, respectively. All samples were diluted 20-fold for dermcidin and left undiluted for chemerin. The defatted colostrum samples were assayed in duplicate. The intra-assay and inter-assay coefficients of variation were 3.2% and 3.5% for dermcidin and 2.8% and 3.5% for chemerin, respectively.

### 4.7. Statistical Analysis

The statistical analysis was performed with TIBCO STATISTICA ver. 13.3 (StatSoft, Inc., Tulsa, OK, USA). Qualitative variables such as the preconception BMI of the mother (normal weight, overweight, obesity), gestational age (near term, term), mode of delivery (vaginal birth, cesarean section) and infant’s gender were presented as frequencies and percentages (% (n/N)). Quantitative variables are shown as the median, mean ± SD (standard deviation) and the twenty-fifth to seventy-fifth percentiles. To compare cohorts, the chi-square test was used. Due to interindividual differences in the biochemical parameters of milk, for analysis, nonparametric tests were used. The milk adipokine concentrations (irisin, obestatin, adropin, visfatin, vaspin, chemerin and dermcidin) are presented as mean ± standard deviation (SD), median and twenty-fifth-seventy-fifth percentiles. Outlier analysis using the Tukey method showed the limits of typical observations, and to reduce model load, the outliers were reduced to limit values. For the calculation of statistical significance, the Kruskal–Wallis test was used. 

The strength and direction of the monotonic relationship between analyzed adipokines and also between milk hormones and maternal and newborn anthropometric variables for GDM and non-GDM (control) groups were estimated according to the Spearman rank correlation and presented in the form of a heat map. Dark red color indicates the strongest positive correlations, and dark green color indicates the strongest negative correlations. A two-tailed *p*-value lower than 0.05 was regarded as significant.

Finally, through the use of multivariate regression models, we went a step further and measured the statistical contribution of the maternal (pre-pregnancy BMI, age and exposure to GDM), obstetrical (gestational age, delivery mode) and neonatal (birth weight, Apgar score) outcomes to colostral adipokine levels.

## 5. Conclusions and Future Perspectives

The level of plasma/serum irisin, obestatin, adropin, visfatin, vaspin, dermcidin and chemerin in diabetes and obesity is well established, in contrast to GDM-affected maternal milk adipokines, which may influence the energy intake, weight gain, growth and development of newborns and infants [57,162,163]. 

Among the analyzed set of molecules, changes in the colostral anti-inflammatory adipokines irisin and vaspin are related to the way of restoring glucose homeostasis in pregnant women. In contrast, managing glucose imbalance during pregnancy did not affect levels of the colostral anti-inflammatory adipokines obestatin and adropin, or the pro-inflammatory adipokines visfatin, chemerin and dermcidin. For the overall GDM group, we did not observe disturbances in the levels of analyzed anti- and pro-inflammatory adipokines, in comparison to the non-GDM group. This might be the net result of early screening diagnosis and implementation of effective treatment of hyperglycemia in pregnant women. 

The mentioned differences between our results and previous data in concentrations of some analyzed milk adipokines might be a consequence of heterogeneous anthropometric characteristics of mothers, such as BMI [65], as well as different geographical zones and other protocols for sampling of milk, and finally the size of cohorts enrolled for the study. Our study may contribute to the development of targeted interventions to optimize the health outcomes for infants born to mothers with GDM. The data presented in our research are an important contribution to the understanding of the potential metabolic programming of future generations by milk adipokines involved in feeding patterns, appetite regulation and infant growth. 

We believe that further research will shed more light on the postnatal development of offspring exposed to maternal GDM during prenatal life. To evaluate the role of milk adipokines as bioactive molecules, further well-organized studies with a longitudinal follow-up are needed. These studies should especially emphasize bigger-size cohorts, analyzing the results in relation to maternal HbA1c values. Moreover, a comparison of anthropometric parameters of children breastfed by GDM and non-GDM mothers should be performed. The potential impact of maternal nutrition and physical activity during pregnancy on milk adipokine levels should be considered. 

## Figures and Tables

**Table 1:** Characteristics of the study population.

	Overall N = 60 % (n/N)	GDM-G1 N = 17 % (n/N)	GDM-G2 N = 17 % (n/N)	Non-GDM N = 26 % (n/N)	Chi-Square Test χ^2^	*p*-Value
Race/ethnicity white Europeans	100% (60/60)	100% (17/17)	100% (17/17)	100% (26/26)	-	-
Day of lactation (mean ± SD) 1st–3rd 4th–7th	2.95 ± 1.76 65.00% (39/60) 35.00% (21/60)	3.41 ± 1.94 58.82% (10/17) 41.18% (7/17)	2.88 ± 1.53 70.59% (12/17) 29.41% (5/17)	2.69 ± 1.78 65.38% (17/26) 34.62% (9/26)	0.52	0.77
Maternal age (mean ± SD) 20–29 30–34 35–40 40+	33.00 ± 4.81 25.00% (15/60) 36.67% (22/60) 33.33% (20/60) 5.00% (3/60)	33.00 ± 4.69 35.29% (6/17) 35.29% (6/17) 23.53% (4/17) 5.88% (1/17)	34.12 ± 5.35 17.65% (3/17) 29.41% (5/17) 47.06% (8/17) 5.88% (1/17)	32.27 ± 4.57 23.08% (6/26) 42.31% (11/26) 30.77% (8/26) 3.85% (1/26)	3.23	0.78
Maternal pre-pregnancy BMI, kg/m^2^ (mean ± SD) underweight (<18.5) normal weight (18.5–24.9) overweight (25.0–29.9) obesity class 1 (30.0–34.9) obesity class 2 (35.0–39.9)	23.99 ± 4.81 3.33% (2/60) 65.00% (39/60) 16.67% (10/60) 11.67% (7/60) 3.33% (2/60)	22.99 ± 4.16 5.88% (1/17) 64.71% (11/17) 23.53% (4/17) 5.88% (1/17) 0.00% (0/17)	27.18 ± 5.79 0.00% (0/17) 41.18% (7/17) 29.41% (5/17) 17.65% (3/17) 11.76% (2/17)	22.55 ± 3.51 3.85% (1/26) 80.77% (21/26) 3.85% (1/26) 11.54% (3/26) 0.00% (0/26)	14.17	0.08
Gestational age (mean ± SD) near term: 35–37 weeks term: 38–41 weeks	38.68 ± 1.33 21.67% (13/60) 78.33% (47/60)	38.77 ± 1.35 23.53% (4/17) 76.47% (13/17)	38.12 ± 1.11 29.41% (5/17) 70.59% (12/17)	39.00 ± 1.38 15.38% (4/26) 84.62% (22/26)	1.24	0.54
Delivery mode vaginal birth cesarean section	26.67% (16/60) 73.33% (44/60)	23.53% (4/17) 76.47% (13/17)	29.41% (5/17) 70.59% (12/17)	26.92% (7/26) 73.08% (19/26)	0.15	0.93
Birth weight (g) (mean ± SD) Appropriate for gestational age	3267.08 ± 547.12 100% (60/60)	3238.82 ± 587.37 100% (17/17)	3187.94 ± 484.11 100% (17/17)	3337.31 ± 571.26 100% (26/26)	-	-
Apgar score [5 min] 6 7 8 9 10	9.64 ± 0.79 1.67% (1/60) 1.67% (1/60) 5.00% (3/60) 10.00% (6/60) 81.67% (49/60)	9.35 ± 1.22 5.88% (1/17) 5.88% (1/17) 5.88% (1/17) 11.76% (2/17) 70.59% (12/17)	9.82 ± 0.53 0.00% (0/17) 0.00% (0/17) 5.88% (1/17) 5.88% (1/17) 88.24% (15/17)	9.81 ± 0.49 0.00% (0/17) 0.00% (0/17) 3.85% (1/26) 11.54% (3/26) 84.62% (22/26)	5.96	0.65
Newborn’s gender male female no information	43.33% (26/60) 50.00% (30/60) 6.67% (4/60)	58.82% (10/17) 35.29% (6/17) 5.88% (1/17)	52.94% (9/17) 47.06% (8/17) 0.00% (0/17)	26.92% (7/26) 61.54% (16/26) 11.54% (3/26)	6.45	0.17

BMI—preconceptional body mass index.

**Table 2:** Concentrations of adipokines in colostrum from diabetic mothers.

Colostral Hormones	Group				* p-Value			
	GDM N = 34	GDM-G1 N= 17	GDM-G2 N = 17	Non-GDM N = 26	GDM vs. Non-GDM	G1 vs. Non-GDM	G2 vs. Non-GDM	G1 vs. G2
Irisin [µg/mL]	22.51 ± 11.42 19.87 (14.80–26.95)	18.11 ± 5.48 17.59 (14.80–22.77)	26.90 ± 14.07 26.95 (15.44–36.99)	21.16 ± 13.39 18.53 (8.82–33.44)	0.53	0.78	0.18	0.04
Adropin [ng/mL]	0.66 ± 0.71 0.49 (0.05–0.92)	0.80 ± 0.83 0.70 (0.06–1.05)	0.52 ± 0.55 0.42 (0.05–0.88)	0.48 ± 0.52 0.34 (0.04–0.91)	0.30	0.18	0.67	0.31
Obestatin [ng/mL]	0.31 ± 0.23 0.28 (0.13–0.39)	0.22 ± 0.16 0.14 (0.12–0.37)	0.39 ± 0.25 0.36 (0.20–0.54)	0.29 ± 0.16 0.30 (0.14–0.38)	0.94	0.22	0.30	0.05
Visfatin [ng/mL]	1.38 ± 0.47 1.38 (1.04–1.73)	1.52 ± 0.47 1.65 (1.15–1.90)	1.25 ± 0.45 1.19 (0.89–1.62)	1.45 ± 0.47 1.30 (1.14–1.69)	0.62	0.65	0.20	0.08
Vaspin [ng/mL]	4.92 ± 3.07 4.69 (2.69–5.58)	3.98 ± 1.76 4.11 (2.23–5.42)	5.80 ± 3.77 4.77 (3.09–7.21)	3.35 ± 1.67 3.12 (2.22 -4.41)	0.06	0.29	0.04	0.31
Chemerin [ng/mL]	1.23 ± 0.84 0.90 (0.54–1.67)	1.14 ± 0.97 0.57 (0.47–2.14)	1.33 ± 0.69 1.10 (0.87–1.67)	1.32 ± 0.93 1.26 (0.62–1.91)	0.88	0.53	0.71	0.21
Dermcidin [ng/mL]	134.49 ± 42.28 136.55 (104.58–165.36)	130.87 ± 39.92 134.51 (104.58–159.64)	138.11 ± 45.45 138.58 (105.22–182.55)	124.03 ± 41.88 124.70 (96.56–156.09)	0.39	0.57	0.38	0.66

Values are given as mean ± SD, median, and twenty-fifth–seventy-fifth percentiles in parentheses. * Kruskal–Wallis test was used for statistical calculations, and a *p*-value lower than 0.05 was regarded as significant.

**Table 3:** Correlations value between the concentration of irisin and other adipokines in milk collected from GDM-G1, GDM-G2, and non-GDM mothers and day of lactation, age, preconceptional BMI, and week of gestation.

	Irisin [µg/mL]		
	GDM-G1	GDM-G2	non-GDM
Day of lactation	−0.27	0.59	0.03
Maternal age [years]	−0.20	0.26	−0.04
BMI [kg/m^2^]	−0.10	0.26	0.45
HBD [week]	0.23	0.05	0.16
Birth weight [g]	−0.08	−0.07	0.40
Chemerin [ng/mL]	0.34	0.03	0.17
Irisin [µg/mL]	-	-	-
Visfatin [ng/mL]	−0.33	0.44	0.20
Vaspin [ng/mL]	0.42	0.20	0.11
Dermcidin [ng/mL]	0.17	−0.51	−0.53
Obestatin [ng/mL]	−0.04	−0.23	0.32
Adropin [ng/mL]	0.17	0.01	0.04

The correlations with statistical significance (*p* < 0.05) are marked red color. BMI—preconceptional body mass index, HBD—week of gestation.

**Table 4:** Associations between colostral adipokine concentrations and maternal factors (β-coefficients and 95% confidence intervals).

Colostral Adipokines	Pre-Pregnancy BMI [kg/m^2^]			Age [Years]			GDM (Ref. Non-GDM)		
	β	95% CI	*p*-Value	β	95% CI	*p*-Value	OR	95% CI	*p*-Value
Chemerin [ng/mL]	−0.13	−0.46–0.20	0.42	−0.12	−0.43–0.19	0.43	0.99	0.44–2.23	0.98
Irisin (µg/mL)	0.40	0.04–0.76	0.03	−0.12	−0.46–0.21	0.46	1.01	0.95–1.07	0.78
Visfatin (ng/mL)	−0.29	−0.70 to 0.12	0.16	−0.14	−0.53–0.24	0.45	1.00	1.00–1.001	0.50
Vaspin (ng/mL)	0.22	−0.11–0.54	0.19	0.55	0.25–0.86	0.0008	1.32	0.93–1.87	0.12
Dermcidin (ng/mL)	−0.07	−0.47–0.33	0.72	−0.04	−0.41–0.34	0.85	1.02	0.99–1.04	0.19
Obestatin (ng/mL)	−0.12	−0.47–0.23	0.49	0.16	−0.17–0.49	0.34	1.00	1.00–1.01	0.50
Adropin (ng/mL)	0.11	−0.20–0.43	0.46	−0.07	−0.37–0.22	0.62	1.00	1.00–1.01	0.16

GDM, gestational diabetes mellitus; β-coefficients and 95% confidence intervals. A *p*-value lower than 0.05 was considered significant.

**Table 5:** Associations between colostral adipokine concentrations and obstetrical/neonatal factors (β-coefficients and 95% confidence intervals).

Colostral Adipokines	Obstetrical Factors						Neonatal Factors					
	Gestational Age (weeks)			Delivery Mode (Ref. Cesarean Section)			Birth Weight [g]			Apgar Score (5 min)		
	β	95% CI	*p*-Value	OR	95% CI	*p*-Value	β	95% CI	*p*-Value	β	95% CI	*p*-Value
Chemerin [ng/mL]	0.14	−0.21–0.49	0.41	1.56	0.57–4.28	0.39	0.16	−0.18–0.51	0.35	0.06	−0.29–0.42	0.72
Irisin (µg/mL)	0.24	−0.14–0.62	0.21	1.00	0.94–1.08	0.91	0.20	−0.18–0.57	0.29	0.21	−0.18–0.59	0.28
Visfatin (pg/mL)	0.25	−0.19–0.69	0.25	1.00	1.00–1.01	0.76	0.35	−0.08–0.78	0.11	−0.07	−0.51–0.37	0.75
Vaspin (ng/mL)	−0.15	−0.50–0.20	0.38	1.60	0.95–2.68	0.08	−0.04	−0.38–0.30	0.81	−0.01	−0.36–0.34	0.96
Dermcidin (ng/mL)	0.45	0.02–0.88	0.04	1.00	0.97–1.03	0.99	0.41	−0.01–0.83	0.06	0.02	−0.41–0.46	0.91
Obestatin (ng/mL)	−0.05	−0.42–0.33	0.80	1.00	1.00–1.01	0.43	0.17	−0.20–0.54	0.36	0.19	−0.19–0.57	0.31
Adropin (ng/mL)	0.01	−0.32–0.35	0.94	1.00	1.00–1.01	0.28	−0.09	−0.42–0.24	0.57	0.14	−0.19–0.48	0.39

GDM, gestational diabetes mellitus; β-coefficients and 95% confidence intervals. A *p*-value lower than 0.05 was considered significant.

## Data Availability

The data underlying this article will be shared on reasonable request to the corresponding author.

## Supplementary Materials

The following supporting information can be downloaded at: https://www.mdpi.com/article/10.3390/ijms26010040/s1.
