# Supplementary material for: Evaluation of Selected Pro- and Anti-Inflammatory Adipokines in Colostrum from Mothers with Gestational Diabetes Mellitus
Source: Int J Mol Sci. 2024 Dec 24;26(1):40. doi: 10.3390/ijms26010040 (PMC11719563; doi:10.3390/ijms26010040)
Supplement: Supplementary file 1 [file ijms-26-00040-s001.zip › ijms-3331303-supplementary.pdf]

Supplementary

Table S1. Correlations value between concentration of adipokine in milk collected from GDM and non-GDM mothers and day of lactation, age, preconceptional BMI and week of gestation.

	Day of lactation	Age [years]	BMI [kg/m²]	HBD [week]	Birth weight [g]	Obestatin [ng/mL]	Adropin [ng/mL]	Visfatin [ng/mL]	Vaspin [ng/mL]	Chemerin [ng/mL]	Dermcidin [ng/mL]
	GDM group										
Day of lactation	1.00										
Maternal age [years]	0.13	1.00									
BMI [kg/m²]	0.10	0.34	1.00								
HBD [week]	-0.24	-0.34	-0.42	1.00							
Birth weight [g]	-0.14	-0.09	-0.21	0.60	1.00						
Obestatin [ng/mL]	-0.36	0.11	0.03	-0.04	0.19	1.00					
Adropin [ng/mL]	-0.10	-0.14	0.04	0.19	0.03	0.08	1.00				
Visfatin [ng/mL]	0.43	-0.05	-0.17	0.14	0.04	-0.71	-0.19	1.00			
Vaspin [ng/mL]	0.02	0.21	0.14	0.00	0.22	0.14	-0.02	-0.02	1.00		
Chemerin [ng/mL]	-0.16	-0.16	-0.09	0.10	-0.05	0.15	0.13	-0.23	0.35	1.00	
Dermcidin [ng/mL]	-0.42	-0.07	0.05	0.28	0.37	0.56	0.10	-0.54	-0.00	-0.05	1.00
	non-GDM group										
Day of lactation	1.00										
Age [years]	-0.02	1.00									
BMI [kg/m²]	0.26	0.33	1.00								
HBD [week]	0.46	-0.18	-0.17	1.00							
Birth weight [g]	-0.09	-0.13	-0.09	0.56	1.00						
Obestatin [ng/mL]	-0.32	0.02	0.25	-0.13	-0.06	1.00					
Adropin [ng/mL]	-0.24	-0.15	-0.10	0.05	-0.12	0.19	1.00				
Visfatin [ng/mL]	0.40	-0.12	-0.00	-0.16	0.18	-0.02	-0.39	1.00			
Vaspin [ng/mL]	0.14	0.35	0.43	-0.01	-0.04	0.05	-0.20	-0.22	1.00		
Chemerin [ng/mL]	-0.39	-0.09	-0.04	0.20	0.16	-0.12	0.11	0.05	0.02	1.00	
Dermcidin [ng/mL]	-0.14	0.33	-0.05	-0.02	-0.31	-0.16	-0.12	-0.35	0.23	-0.03	1.00

The correlations with statistical significance (p<0.05) are shown in red. BMI- preconceptional body mass index, HBD- week of gestation
